# The sleeping brain’s connectivity and family environment: characterizing sleep EEG coherence in an infant cohort

**DOI:** 10.1038/s41598-023-29129-3

**Published:** 2023-02-04

**Authors:** Andjela Markovic, Sarah F. Schoch, Reto Huber, Malcolm Kohler, Salome Kurth

**Affiliations:** 1 Department of Psychology, University of Fribourg, Fribourg, Switzerland; 2 Department of Pulmonology, University Hospital Zurich, Zurich, Switzerland; 3 University Hospital of Child and Adolescent Psychiatry and Psychotherapy, University of Bern, Bern, Switzerland; 4 Center of Competence Sleep & Health Zurich, University of Zurich, Zurich, Switzerland; 5 Donders Institute for Brain, Cognition and Behaviour, Radboud University Medical Centre, Nijmegen, The Netherlands; 6 Child Development Center, University Children’s Hospital Zurich, Zurich, Switzerland; 7 Department of Child and Adolescent Psychiatry and Psychotherapy, Psychiatric Hospital, University of Zurich, Zurich, Switzerland

**Keywords:** Neuroscience, Sleep

## Abstract

Brain connectivity closely reflects brain function and behavior. Sleep EEG coherence, a measure of brain’s connectivity during sleep, undergoes pronounced changes across development under the influence of environmental factors. Yet, the determinants of the developing brain’s sleep EEG coherence from the child’s family environment remain unknown. After characterizing high-density sleep EEG coherence in 31 healthy 6-month-old infants by detecting strongly synchronized clusters through a data-driven approach, we examined the association of sleep EEG coherence from these clusters with factors from the infant’s family environment. Clusters with greatest coherence were observed over the frontal lobe. Higher delta coherence over the left frontal cortex was found in infants sleeping in their parents’ room, while infants sleeping in a room shared with their sibling(s) showed greater delta coherence over the central parts of the frontal cortex, suggesting a link between local brain connectivity and co-sleeping. Finally, lower occipital delta coherence was associated with maternal anxiety regarding their infant’s sleep. These interesting links between sleep EEG coherence and family factors have the potential to serve in early health interventions as a new set of targets from the child’s immediate environment.

## Introduction

Effective integration of information across different brain regions is essential to brain function^1^. Imaging studies propose brain connectivity as a marker of cognitive development^2^. While the early maturation of brain structure organization (e.g., by neuronal migration and differentiation) is largely genetically determined^3^, the ultimate patterns of synaptic connectivity and communication between brain regions are affected by environmental input^4,5^. We therefore set out to identify external factors that contribute to brain connectivity during the first year of life. Insights in these processes may open new avenues for early interventions by targeting a child’s immediate environment.

Brain network activity is organized through both short-range (i.e., interactions between adjacent regions) and long-range (i.e., interactions between distant regions) connectivity^6^. The balance between these two types of connectivity may be one particular determinant of healthy brain development^7^. Early in life, functional imaging reveals a gradual shift from short-range connectivity towards long-distance communication with increasing age (e.g.^8,9^). This shift is likely promoted by an increase in myelin content which leads to a widely connected mature brain^10^. Across maturation, infancy has been proposed as the most critical phase for development and organization of brain network activity. Disturbances during this period may lead to far-reaching consequences such as neuropsychiatric disorders later in life (reviewed in Ref.^11^).

Electroencephalography (EEG) is the gold standard for assessing brain activity during sleep, a special state of body and mind. Emerging evidence suggests that sleep actively contributes to brain’s maturational processes including cortical plasticity critically shaping cognitive development (reviewed in Ref.^12^). Sleep EEG coherence, a widely used measure of functional connectivity in sleep research, increases during childhood and adolescence, with most pronounced changes in the delta and sigma bands^13,14^. The delta band encompasses slow wave activity (i.e., high-amplitude low-frequency oscillations occurring during deep sleep), while the sigma band encompasses sleep spindles, waxing and waning oscillatory events. Thalamo-cortical circuits play a crucial role in generating both slow waves and sleep spindles, which represent the key electrophysiological characteristics of non-rapid eye movement (NREM) sleep^15^. In the first year of life, these oscillations have been associated with motor and cognitive development^16,17^. Yet, the characteristics of sleep EEG coherence across this developmental period are largely understudied.

The changes to sleep EEG coherence observed during childhood^13^ and adolescence^14^ may reflect increased myelination and the associated improvement in brain efficiency^10^. This notion is in line with a recent concept proposing one core role of sleep to be brain myelin maintenance^18^. Indeed, the rate of increase in sigma coherence is positively associated with improvement in cognitive performance during adolescence^19^. For the same developmental period, a twin study revealed that despite high intra-individual stability of sleep EEG coherence across multiple nights, this measure is strongly affected by environmental factors. In this study, 66% of the variance could be explained by the environment, while genes accounted for only 19%^20^. Nevertheless, the precise nature of such environmental factors remains unknown and specific investigations to identify the adverse and protective determinants of sleep EEG coherence have so far been lacking.

Adverse events early in life can have severe consequences for development. To the best of our knowledge, only one study examined their effects on EEG coherence. In this interventional study, institutionalized children in Romania were placed into foster care and demonstrated a better cognitive outcome at 42 and 54 months of age in comparison to children who remained in the institution^21^. Importantly, children placed into foster care before 2 years of age demonstrated the best response to the intervention in terms of cognitive development, showing early childhood as a sensitive developmental period. Crucially, for children placed into family care environments, earlier age at foster care placement was associated with lower wake EEG short-distance coherence in the alpha and beta bands at the age of 3.5 years^22^. Taken together, a challenging environmental context during infancy and early childhood (e.g., reduced caregiver support and stimulation) may negatively impact brain development and behavioral outcomes later in life. However, whether more subtle differences in parenting and family context can affect EEG coherence in early life remains unknown.

The current study thus aims to (1) characterize sleep EEG coherence in the delta and sigma bands, frequencies undergoing most pronounced developmental changes in coherence^13,14^, and (2) identify modifiable environmental factors that affect sleep EEG coherence at the age of 6 months. We analyzed three sleep-related factors within the family context: (1) caregiver-determined infant sleep habits, (2) caregivers’ principles regarding structure and regularity of their infant’s sleep, and (3) caregivers’ anxiety and concerns about their infant’s sleep. Introducing regular sleep rhythms and bedtime routines has proven effective in managing sleep disorders in children^23^ and thus represents a powerful early intervention. Furthermore, previous studies suggest that maternal stress and anxiety during antenatal and perinatal periods are associated with the child’s neurodevelopment after birth^24^ as well as later cognitive, behavioral and emotional problems^25^. Such observations highlight the importance of interventional programs to mitigate maternal stress during pregnancy^25^.

Based on these observations, we hypothesize that more structure and regularity imposed by the caregivers, as well as low anxiety and concern about infant sleep will be associated with increased long-range (i.e., between regions) and decreased short-range (i.e., within region) coherence. We thereby operationalize the dominance of long-range over short-range connections as a maturational marker according to the abovementioned work in neuroimaging^8^.

## Methods

### Participants

Nighttime sleep EEG was recorded in 35 infants as part of a longitudinal study examining the association between sleep and behavioral development in the first year of life^26^. Data from 4 participants were excluded due to inability to fall asleep (N = 2) or insufficient signal quality (N = 2) yielding sleep EEG recordings from 31 infants aged 5.5 to 7.4 months (mean age = 5.9 ± 0.5 months; 15 females) that were analyzed. All infants were healthy, primarily breastfed (> 50% of feedings), and received no antibiotics until age 3 months. They were vaginally delivered after 37 to 43 weeks of gestation with a birth weight above 2500 g. Study procedures were approved by the cantonal ethics committee (BASEC 2016–00,730) and adhered to the declaration of Helsinki. Written informed consent was obtained from the parents.

### Sleep EEG

Sleep EEG recordings were conducted at participants’ homes and scheduled to individual infants’ habitual evening bedtime. Up to 2 h of sleep were recorded with a 124-channel sponge electrode net (Electrical Geodesics Sensor Net, Electrical Geodesics Inc., EGI, Eugene, OR) at a sampling rate of 500 Hz applying a bandpass filter at 0.01–200 Hz. Impedances were kept below 50 kΩ. For analysis, data were bandpass filtered at 0.5–50 Hz and downsampled to 128 Hz. Sleep stages were scored in 20-s epochs according to the AASM Manual^27^ with consensus agreement between two independent scorers. Epochs with artifacts were excluded using a semiautomated procedure whenever power exceeded a threshold in the low (0.75–4.5 Hz) and high (20–30 Hz) frequency ranges^28^. After excluding the outermost channels as well as channels with poor signal quality, each channel’s signal was recalculated relative to the average of all channels (i.e., average reference). The maximal available duration of artifact-free NREM sleep across all infants (i.e., the first 80 20-s epochs amounting to 26.7 min) was analyzed.

### Coherence

Coherence was calculated between all possible 5886 channel pairs as $\frac{{\left|{P}_{xy}(f)\right|}^{2}}{{P}_{xx}(f){P}_{yy}(f)}$ where ${P}_{xy}(f)$ is the cross-spectral density and ${P}_{xx}(f)$ and ${P}_{yy}(f)$ are the auto-spectral density functions of the two signals $x$ and $y$ at frequency $f$^29^. We applied Welch’s method for 20-s epochs (Hanning window; no overlap; frequency resolution 0.25 Hz) in MATLAB (The MathWorks Inc., Natick MA, USA) in the delta (0.75–4.25 Hz) and sigma (9.75–14.75 Hz) bands.

To reduce the amount of data and preserve essential information as well as the spatial context, we used a data-driven clustering approach to identify biologically relevant regions of interest, referred to as functional units^30^. This approach only considers coherence values above a predefined threshold as meaningful and takes into account the spatial constellation of electrodes. After averaging coherence across subjects, the FuMapLab toolbox^31^ in MATLAB was used to partition NREM sleep delta and sigma coherence into functional units. Partitioning is based on the level of coherence of each electrode with its neighboring electrodes and compares this value to the predefined coherence threshold. The procedure starts by assigning each electrode to a separate functional unit. A method is applied based on watershed algorithms typically used in image processing to detect boundaries between different segments^32^. This method merges single functional units if they are spatially connected and if their union is a clique (i.e., a set of electrodes in which every pair of electrodes is connected with their coherence exceeding the predefined threshold). For the final set of functional units, the coherence between units is compared to the predefined threshold thereby allowing for an examination of both within- as well as between-region coherence. Within- and between-region coherence were used as a proxy for short-range and long-range connectivity. We acknowledge that, due to the data-driven nature of this approach, the selected within- and between-region coherence may not capture the absolute difference between short and long distances. We applied a minimal functional unit size (i.e., number of electrodes) of 5 and a threshold for coherence of 0.5 to best address the trade-off of markedly reducing the amount of data while preserving their spatial information. Further information about the clustering algorithm is provided as Supplementary Methods.

As coherence between electrodes with very small (< 10 cm)^33,34^ or very large (> 20 cm)^35^ spatial distances can artificially be inflated due to volume conduction resulting from different electrical properties of brain tissues, we calculated current source density (CSD) estimates from our EEG signals. We used the CSD toolbox^36^ in MATLAB, an implementation of a spherical spline algorithm^37^, to compute CSD estimates and account for volume conduction effects. This transformation estimates the current from the underlying neuronal generators based on the EEG signals recorded from the scalp, and is independent of the recording reference^38^. The approach reduces redundancy without losing relevant information from the EEG signals^38^. For statistical analyses, we averaged coherence of CSD estimates for all detected functional units and their connections. Only CSD-based coherence is presented throughout the paper.

### Familial factors

To identify sleep-related environmental factors associated with infant sleep EEG coherence, we selected potentially modifiable items from the Brief Infant Sleep Questionnaire^39^, Baby Care Questionnaire^40^, and the Maternal Cognitions about Infant Sleep Questionnaire^41^. All questionnaires were completed by the mother prior to or after the EEG assessment.

#### Brief Infant Sleep Questionnaire

From the Brief Infant Sleep Questionnaire, we selected those items that specifically reflect parental practices regarding their child’s sleep and thus behavior that could in general be modified with a targeted intervention: infant’s sleeping arrangement (i.e., own bed in his/her own room, own bed in parents’ room, parents’ bed, own bed in sibling’s room, other) and bedtime routine regularity (i.e., on how many days does the child have the exact same bedtime routine in a typical week).

#### Baby Care Questionnaire

From the Baby Care Questionnaire, scores for the two subscales, Structure and Attunement, were calculated for all nine sleep-related items. Structure reflects how strongly the parents value regularity and routines in parenting principles, while Attunement mirrors the parents’ willingness to flexibly react to their child’s cues^40^. Items were rated on a 4-point scale ranging from “strongly disagree” (1) to “strongly agree” (4). The subscale score represents the mean across all scored subscale items. The original questionnaire was translated to German by the authors.

#### Maternal Cognition about Infant Sleep Questionnaire

From the Maternal Cognition about Infant Sleep Questionnaire, we calculated the total score based on all subscale scores^41^. The subscales address Limit Setting, Anger, Doubt, Feeding, and Safety. High scores in Limit Setting indicate maternal difficulty to resist her child’s demands during night. For Anger, high scores reflect maternal experience of negative feelings in response to her child’s demands during night. High scores for Doubt indicate maternal doubt about parenting competence regarding the child’s demands during night. High scores on the Feeding subscale indicate maternal beliefs that nocturnal feedings are important to soothe the infant. Finally, high scores on the Safety subscale indicate maternal concerns about the child’s safety during night. Items were rated on a 6-point scale ranging from “strongly disagree” (1) to “strongly agree” (6).

### Statistical analyses

We examined the association between sleep EEG coherence and familial factors by means of linear regression models. First, we used least angle regression^42^, a model selection algorithm, to identify the relevant factors out of the following: age, sex, sleeping arrangement, bedtime routine, structure, attunement, maternal cognitions, and frequency of breastfeeding. The frequency of breastfeeding (i.e., never, rarely, occasionally, regularly, daily) was included based on previous reports showing associations of breastfeeding duration with infants’ sleeping arrangement^43^ as well as the development of brain connectivity^44,45^. For each of the detected functional units and connections in the delta and sigma bands, we computed the model selected by means of least angle regression with coherence as the outcome variable. P-values were corrected by means of false discovery rate^46^ to account for multiple testing (i.e., the number of applied models). For each model, we calculated McFadden’s R^2^^47^ to assess the explained variance in comparison to the null model. The statistical analyses were conducted in R^48^. All figures were created in MATLAB.

## Results

**Figure 1:**
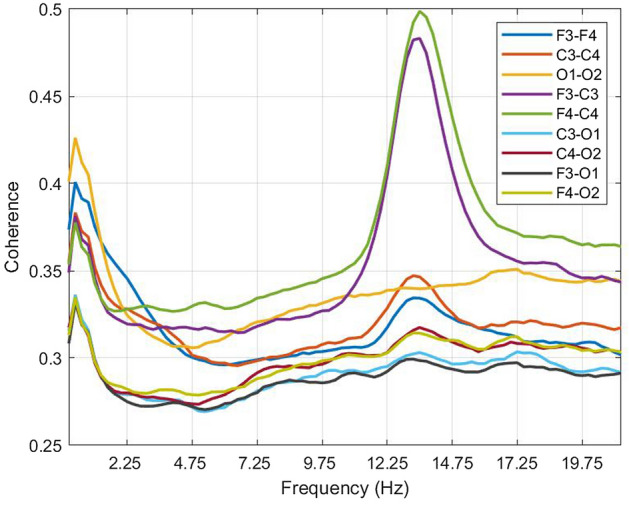
Non-rapid eye movement (NREM) sleep coherence spectra for a subset of connections averaged across 31 infants. The connections were chosen to ensure comparability to previously published work regarding sleep EEG coherence in children^13^ and adolescents^14^ and are illustrated in different colors.

Coherence of the infant sleep EEG showed prominent peaks in the delta and sigma frequency ranges of the spectrum (Fig. 1), comparable to existing work on sleep EEG coherence in childhood^13^ and adolescence^14^. Particularly the intra-hemispheric connections between frontal and central electrodes (i.e., F3-C3 and F4-C4), showed high levels of coherence, followed by inter-hemispheric connections over occipital (O1–O2), central (C3–C4), and frontal (F3–F4) regions. Similar to sleep EEG coherence in 2-year-olds^13^, no sigma peak was observed in the inter-hemispheric occipital connection (O1–O2).

**Figure 2:**
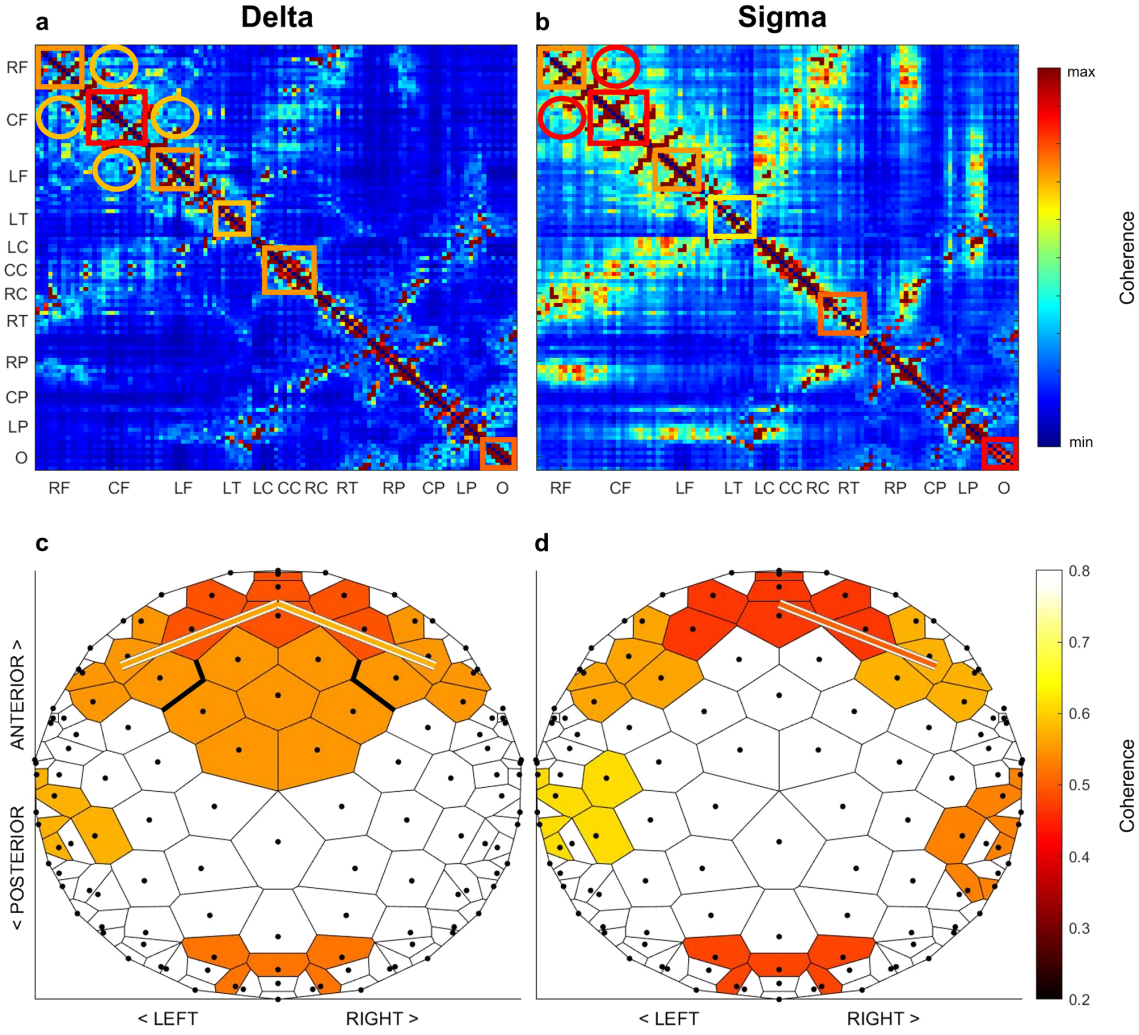
Non-rapid eye movement (NREM) sleep EEG coherence at 6 months of age. Panels (**a,b**) show heatmaps of NREM sleep delta and sigma coherence averaged across 31 infants. Complete coherence data from 5886 connections were grouped into the following regions: right frontal (RF), central frontal (CF), left frontal (LF), left temporal (LT), left central (LC), central central (CC), right central (RC), right temporal (RT), right parietal (RP), central parietal (CP), left parietal (LP), occipital (O). Cool colors (blue) represent lower coherence, while warm colors (red) represent greater coherence. Colored squares correspond to color-matched functional units shown in (**c,d**). Circles depict the connections corresponding to the lines between the units in (**c,d**). The units and their connections were detected through a data-driven clustering approach, such that regions exhibiting the greatest levels of coherence were detected. Colors depict the level of coherence averaged across subjects with brighter color tones indicating higher values. Same-color units are separated by means of bold lines.

To evaluate regional aspects, we examined all pairs of electrodes (Fig. 2a,b) and found a similar regional pattern in both bands, albeit with greater coherence in the sigma band. Adjacent regions demonstrated the highest levels of coherence, particularly in frontal areas (upper left corner in Fig. 2a,b). Additionally, we found strong connections between frontal and central regions followed by central and parietal, frontal and parietal, as well as parietal and occipital regions (Fig. 2a,b). Interestingly, strong connections were predominantly found within the same hemisphere (Fig. 2a,b).

Next, we identified clusters of electrodes demonstrating the highest level of coherence. The clustering method by ten Caat^31^ resulted in six functional units in both delta and sigma bands with greatest coherence over frontal regions (0.68 to 0.81; Fig. 2c,d). Between-region coherence exceeded the threshold of 0.5 between the frontal unit and the two neighboring units in the delta band (left = 0.56 and right = 0.58), and between the frontal and the right neighboring unit in the sigma band (0.5). These connections are depicted through lines connecting functional units in Fig. 2 (c, d). This indicates that sleep EEG coherence in infants at age 6 months has a strong topographical domain with a frontal focus in both delta and sigma bands.

**Figure 3:**
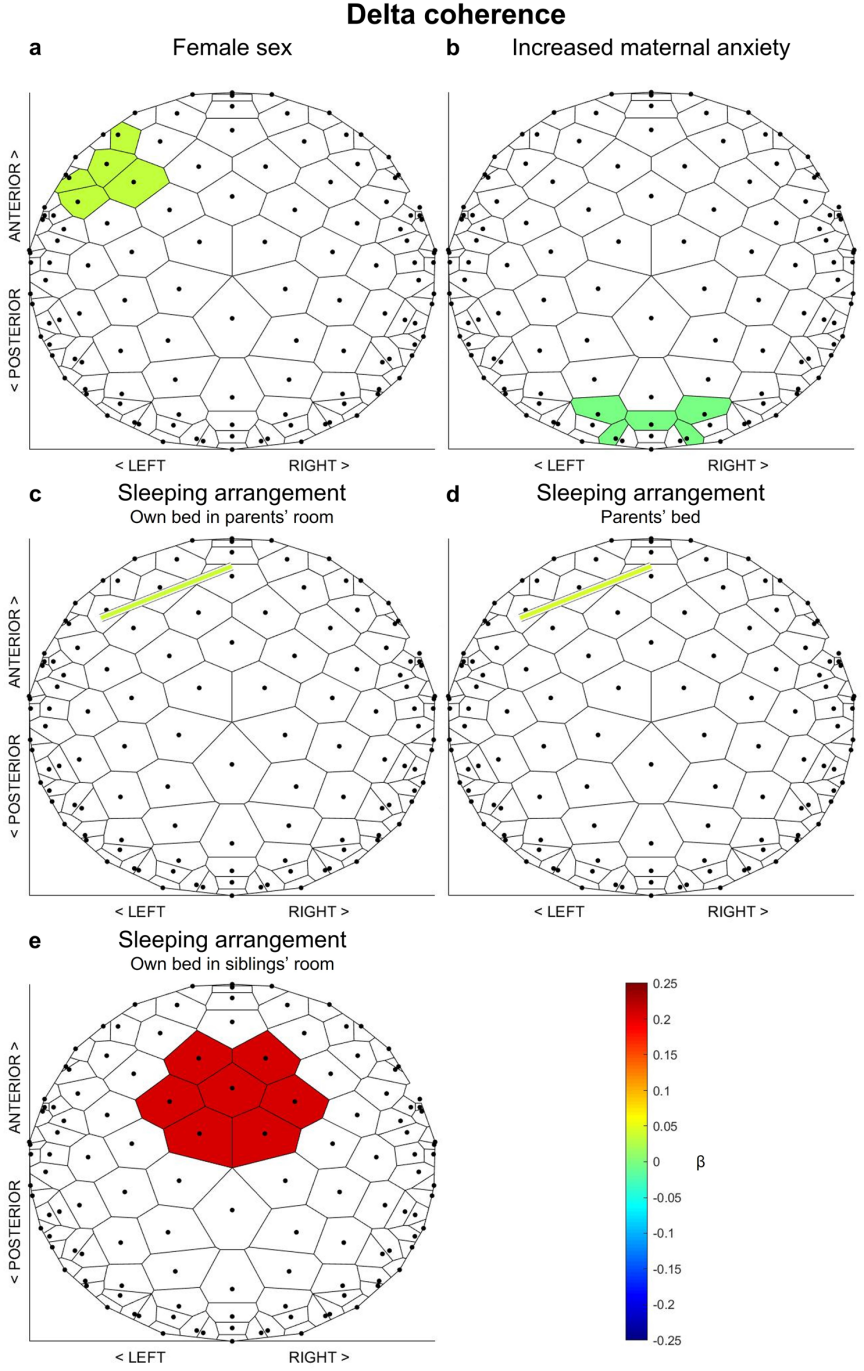
Significant associations of non-rapid eye movement (NREM) sleep delta coherence with sex (**a**) and familial factors (**b** to **e**) resulting from linear regression models. Only effects that remained significant after correcting for multiple comparisons by means of false discovery rate are shown. Colors represent the magnitude of the association as reflected in unstandardized beta coefficients. All associations were positive except for maternal anxiety where lower coherence was associated with increased maternal anxiety (**b**).

Finally, for the detected units and their inter-connections, we quantified the association of sleep EEG coherence in the delta and sigma bands with age, sex, frequency of breastfeeding, and familial factors. The distribution of these factors across infants (Table 1) is in the expected range with the exception of maternal cognition scores which are slightly higher than previously reported for mothers of healthy children^41,49^. However, we note the older age of the children in these reports (i.e., up to 3 years) as compared to the current sample and believe that the natural increase in maternal anxiety and concerns in the first months after birth may be reflected in our data. In the delta band, girls demonstrated increased coherence compared to boys within the left frontal unit (b = 0.032, p = 0.007; Table 2, Fig. 3a). Furthermore, maternal anxiety regarding their infants’ sleep was associated with lower delta coherence within infants’ occipital unit (b =  − 0.002, p = 0.007; Table 2, Fig. 3b). Additionally, co-sleeping arrangements were associated with greater coherence such that infants sleeping in their own bed in the parents’ room (b = 0.037, p = 0.003; Table 2, Fig. 3c) or in the parents’ bed (b = 0.043, p = 0.010; Table 2, Fig. 3d) had greater coherence between the left and the central frontal unit, and those sleeping in a room with their sibling(s) had greater coherence within the central unit (b = 0.211, p = 0.011; Table 2, Fig. 3e) compared to other sleeping arrangements. No significant associations were found for infants’ age, frequency of breastfeeding, bedtime routine, or caregivers’ principles regarding structure and regularity of their infants’ sleep (Table 2). These linear regression models improved the explained variance of the null model by 25 to 39% as reflected in McFadden’s R^2^ (Table 2) suggesting a considerable contribution of the selected factors to explaining the inter-individual variability in delta coherence, while leaving room for further unrecognized influences. In the sigma band, none of the factors were selected by means of least angle regression and thus no further models were computed.

**Table 1:** Characteristics of the participants shown as the mean, the standard deviation (SD) and the range (minimum-maximum), or the number (N) across 31 infants.

Characteristic	
Age (months) Mean ± SD (range)	5.9 ± 0.5 (5.5–7.4)
Sex N	15 females
Breastfeeding N	
Daily	29
Never	2
Sleeping arrangement N	
Own bed in own room	8
Own bed in parents’ room	12
Parents’ bed	3
Own bed in sibling’s room	1
Other	7
Bedtime routine Mean ± SD (range)	4.1 ± 0.9 (1–5)
Structure Mean ± SD (range)	2.9 ± 0.4 (2–3.6)
Attunement Mean ± SD (range)	3.1 ± 0.4 (2.5–4)
Maternal cognitions Mean ± SD (range)	35 ± 11 (9–57)

The bedtime routine was rated from 1 to 5 with higher scores representing more regularity. Structure and attunement were rated on a 4-point scale ranging from “strongly disagree” (1) to “strongly agree” (4). Maternal cognitions are represented as the total score of subscale composite scores as described in the original publication^41^ with higher scores indicating greater maternal anxiety and concerns about the infant’s sleep.

**Table 2:** Associations of sleep EEG coherence in the non-rapid eye movement (NREM) sleep delta band with age, sex, frequency of breastfeeding and the familial factors regarding infant sleep habits (sleeping arrangement and bedtime routine), caregivers’ principles regarding structure and regularity of their infant’s sleep (structure and attunement), and caregivers’ anxiety regarding their infant’s sleep (maternal cognitions).

Factor	Delta coherence							
	Left frontal	Central frontal	Right frontal	Left to central frontal	Right to central frontal	Central	Left temporal	Occipital
	β (p)	β (p)	β (p)	β (p)	β (p)	β (p)	β (p)	β (p)
Age	**–**	**–**	**–**	**–**	**–**	**–**	**–**	**–**
Sex	**0.032 (0.007)**	**–**	0.013 (0.114)	**–**	**–**	**–**	**–**	**–**
Breastfeeding	**–**	**–**	**–**	**–**	**–**	**–**	**–**	**–**
Sleeping arrangement								
Own bed in own room	**–**	0.012 (0.584)	**–**	0.015 (0.234)	**–**	0.037 (0.347)	**–**	**–**
Own bed in parents’ room	**–**	0.038 (0.074)	**–**	**0.037 (0.003)**	**–**	0.043 (0.238)	**–**	**–**
Parents’ bed	**–**	0.049 (0.103)	**–**	**0.043 (0.010)**	**–**	0.084 (0.100)	**–**	**–**
Own bed in sibling’s room	**–**	0.028 (0.535)	**–**	0.022 (0.362)	**–**	**0.211 (0.011)**	**–**	**–**
Bedtime routine	**–**	**–**	**–**	**–**	**–**	**–**	**–**	**–**
Structure	**–**	**–**	0.026 (0.103)	**–**	**–**	**–**	**–**	**–**
Attunement	− 0.037 (0.129)	**–**	0.037 (0.036)	**–**	**–**	**–**	**–**	**–**
Maternal cognitions	**–**	− 0.001 (0.437)	**–**	**–**	**–**	**–**	**–**	** − 0.002 (0.007)**
McFadden’s R^2^	0.27	0.27	0.37	0.39	0	0.28	0	0.25

Sex was coded such that positive coefficients represent larger values in females. For the sleeping arrangement, the category “Other” served as the reference. Unstandardized beta coefficients (b) and uncorrected p-values (p) from linear regression models are shown. For factors that did not survive selection, these values are missing.

Effects that remained significant after correcting for multiple comparisons by means of false discovery rate are bolded. The last row shows McFadden’s R^2^ indicating that the models improved the explained variance of the null model by 25 to 39%.

## Discussion

We examined NREM sleep EEG coherence as a measure of neuronal connectivity in healthy 6-month-old infants. First, we applied a clustering method to identify functionally meaningful groups of electrodes and found that regions with greatest coherence were located over frontal areas in both delta and sigma bands. Second, we investigated links of coherence to familial factors within and between these clusters including (1) infant sleep habits, (2) caregivers’ principles regarding structure and regularity of their infant’s sleep, and (3) caregivers’ anxiety and concern about their infant’s sleep. We observed greater delta coherence over central and frontal regions in co-sleeping infants, and lower occipital delta coherence in infants with more concerned mothers.

Our results indicate interesting associations of sleep EEG coherence with aspects of familial context. We found that infants in an environment of increased maternal concerns showed lower occipital delta coherence, confirming our hypothesis and agreeing with previous reports that maternal stress is a determinant of their children’s neurodevelopment^24^. Specifically, maternal stress and anxiety during pregnancy have been associated with decreased fetal cerebellar-insular^50^ as well as parieto-frontal and occipital^51^ resting-state functional connectivity in utero. Of note, children of anxious and stressed women show increased wakefulness in late pregnancy^52^ and increased sleep concerns in toddlerhood^50^. These observations possibly indicate a disruption of circadian rhythms potentially mediated through elevated cortisol levels and thereby altered hypothalamo-pituitary-adrenocortical responses^53^. Similar mechanisms may explain the association between occipital delta coherence and maternal concerns in our study. However, as localization of neuronal sources generating the analyzed EEG signals is beyond the scope of this work, we cannot draw any conclusions about the responsible anatomical structures.

In addition, we found that infants whose sleeping arrangement involved contact with other family members exhibited greater delta coherence over central and frontal brain regions. Co-sleeping has been described as an enriched sensory environment^54^ with several positive effects for children’s later developmental outcomes including increased self-regulatory behaviors^55^ and secure attachment^56^. Such effects may be implicated in our finding of increased central and frontal coherence in co-sleeping children. This is not surprising, given that the prefrontal cortex is responsible for functions involved in self-regulation^57^. Moreover, increased functional connectivity in temporo-limbic regions was observed in 9-year-old children with secure attachment in comparison to children with insecure attachment of the same age^58^.

As correlations between maternal stress and co-sleeping have been demonstrated^59^ such that stress may impact the decision to co-sleep and vice versa, novel interventions should target co-sleeping and parental stress in synchrony to develop individualized solutions. Given the observed associations of these factors with sleep EEG coherence, such interventions may not only contribute to a healthy family environment, but they may also support the child’s brain development. Of note, interventions and decisions regarding co-sleeping, a topic of ongoing debate, should consider the current recommendations stating that infants should sleep in the parents’ room on a separate surface at least for the first 6 months of life to reduce the risk of sleep-related infant death^60^. Our findings do not conflict with these recommendations, as they are not restricted to bed-sharing but also apply to room-sharing and thus highlight the importance of proximity to other family members as compared to solitary sleeping.

The presented coherence spectra correspond with observations from studies including preschool children^13^ and adolescents^14^, showing most coherent activity in the delta and sigma frequency ranges. Interestingly, the observed sigma peak in coherence spectra appears to decrease between 2 and 3 years to increase again at age 5 years^13^. This suggests a transition in developmental dynamics of coherence in the frequency range of sleep spindles during preschool years. Similarly, sleep spindle activity changes from 12 to 30 months of age, with shifts in topography and reduced density, duration, and frequency^16^, in line with the model developed by Cao et al.^61^. This computational model suggests a crucial transition in the function of sleep between ages 2 and 3 years: from neural reorganization and learning—to repair and clearance processes. Thus, with this ontogenetic switch in the function of sleep, it is possible that sleep spindles as well as their synchronization serve different purposes in the first year of life compared to preschool age. Furthermore, we found that regions with greatest coherence were primarily located over frontal areas in both delta and sigma bands (Fig. 2c,d). This frontal focus is in agreement with recent work in newborns^62^ showing a strong involvement of frontal brain regions during quiet sleep. Given that frontal regions are involved in cognitive functioning^63^, sleep EEG coherence may be an early index of cognitive neurodevelopment.

Compared to earlier low-resolution studies^13,14^, the current high-density data revealed detailed spatial patterns. These patterns included locally focused coherence showing the strongest connections between adjacent regions and progressively becoming weaker with larger distances. Similarly, the observed associations with familial factors were restricted to short-range connections. We thus found no evidence that the balance between short-range and long-range connectivity may represent a maturational marker at 6 months of age. However, we note that the detected between-region connections were limited to neighboring regions and, therefore, do not exclusively represent long-range connectivity. Indeed, previous reports have shown a dominance of short-range connectivity early in life (e.g.^8,64^), but also that short-range connections may represent an important aspect of brain connectivity throughout life (reviewed in Ref.^7^). Furthermore, we observed greater coherence of within-hemisphere connections compared to connections between the hemispheres (Fig. 2a,b). In line with the interpretation by Tarokh et al.^14^, this may be due to the lower abundance of cortical white matter connecting the hemispheres as compared to white matter connecting regions within a hemisphere^65^. The two hemispheres are connected through the corpus callosum which rapidly grows in the first months after birth^66^. Although callosal myelin content is positively associated with slow wave propagation distance during childhood^67^, the long-range connections between the hemispheres may still be underdeveloped in infancy. Furthermore, as coherence is a measure of functional connectivity, its spatial distribution may demonstrate differences among vigilance states NREM sleep, REM sleep and waking. Indeed, a recent study applying functional near-infrared spectroscopy has reported increased long-range inter-hemispheric connectivity during active sleep (equivalent of REM sleep in newborns) and increased short-range intra-hemispheric connectivity in quiet sleep (newborn equivalent of NREM sleep)^68^. We thus conclude that brain connectivity during NREM sleep at 6 months is driven by propagation of activity through short-range pathways.

A multitude of factors interact in a family environment, making single effects difficult to isolate. Considering the small sample size in our cohort, we cannot exclude the possibility that further interactions between family environment and sleep EEG coherence remained unrecognized due to a lack of statistical power. For example, the current analysis did not identify any factors with a significant contribution to sigma coherence. Although a considerable contribution of environmental factors has been observed for both delta and sigma coherence in adolescence, the environmental impact was more pronounced in the delta band^20^. This may suggest that sigma coherence is not as malleable as delta coherence which is not surprising given that the two types of oscillations reflect different biological processes and undergo unique maturational dynamics^67,69^. While sleep spindles are generated in the thalamus through interactions between thalamic reticular nucleus neurons and thalamocortical neurons to be propagated to the cortex^70,71^, a cortical origin of slow waves as well as a crucial role of cortico-cortical connections for their synchronization have been demonstrated^72^. This may explain differences in the functional as well as the clinical relevance of these oscillations which have repeatedly been reported. For example, sleep spindle density has been identified as a predictor of behavioral outcomes in infancy, whereas slow wave activity showed no such effect^73^. Furthermore, increases in sleep EEG coherence in children with childhood onset schizophrenia as compared to healthy controls were more prominent in the delta than in the sigma band^74,75^. Combined with the current findings, these observations highlight the value of delta coherence as a clinically relevant target for early interventions.

In contrast to previous work^13,14^, the spectral profile of infant coherence in our study did not increase towards higher frequencies in inter-hemispheric connections (e.g., C3–C4), nor decrease in intra-hemispheric connections (e.g., C3-O1). This discrepancy may be due to the difference in developmental stages or the shorter duration of current recordings. Both previous studies used whole-night sleep EEG data to capture across-night changes in coherence that are not captured by the first NREM sleep episode included in our analysis. With regards to coherence as a metric of brain connectivity, it is important to consider volume conduction effects. Such effects artificially distort coherence depending on spatial properties of electrode pairs. However, we took several steps to mitigate this potential limitation. First, we applied average reference which is a good approximation of absolute voltage potentials with high-density EEG data^76^. Second, the clustering method addresses volume conduction effects by considering the spatial constellation of electrodes. Third, we additionally calculated coherence from current source density (CSD) estimates, which largely reduces volume conduction effects^38^.

## Conclusions

A strong contribution of environmental factors to sleep EEG coherence has previously been reported in adolescents^20^. To identify these factors, we examined associations between sleep EEG coherence and sleep-related parenting practices and concerns, and found first evidence for such associations in infancy. Considering the importance of sleep EEG coherence as a non-invasive measure of brain connectivity, our findings have implications for building effective early health interventions targeting the child’s immediate environment.

## Supplementary Information

Supplementary Information.

## Data Availability

The data underlying this article will be shared on reasonable request to the corresponding author.
