# Supplementary material for: The sleeping brain’s connectivity and family environment: characterizing sleep EEG coherence in an infant cohort
Source: Sci Rep. 2023 Feb 4;13:2055. doi: 10.1038/s41598-023-29129-3 (PMC9899221; doi:10.1038/s41598-023-29129-3)
Supplement: Supplementary file 1 — Supplementary Information. [file 41598_2023_29129_MOESM1_ESM.pdf]

The Sleeping Brain's Connectivity and Family Environment: Characterizing Sleep EEG Coherence in an Infant Cohort

Andjela Markovic*[1,2,3], Sarah F. Schoch[2,4,5], Reto Huber[4,6,7], Malcolm Kohler[2,4], Salome Kurth[1,2,4]

[1]Department of Psychology, University of Fribourg, Fribourg, CH

[2]Department of Pulmonology, University Hospital Zurich, Zurich, CH

[3]University Hospital of Child and Adolescent Psychiatry and Psychotherapy, University of Bern, Bern, CH

[4]Center of Competence Sleep & Health Zurich, University of Zurich, Zurich, CH

[5]Donders Institute for Brain, Cognition and Behaviour, Radboud University Medical Centre, Nijmegen, NL

[6]Child Development Center, University Children's Hospital Zurich, Zurich, CH

[7]Department of Child and Adolescent Psychiatry and Psychotherapy, Psychiatric Hospital, University of Zurich, Zurich, CH

*Corresponding author:

Andjela Markovic, PhD

Department of Psychology, University of Fribourg

Rue de Faucigny 2, 1700 Fribourg

Switzerland

andjela.markovic@unifr.ch

Supplementary Methods

We applied a clustering method developed by ten Caat et al. [1] to detect biologically relevant regions of interest (i.e., functional units) relying on an edge-based watershed transform typically used for image segmentation [2]. This approach uses Voronoi diagrams to determine the spatial constellations between electrodes. Such diagrams partition the plane into cells consisting of points that are closer to that cell's vertex (i.e., electrode) than to any other vertex [3]. Voronoi neighbors are defined as two cells that have a common boundary. Pseudocode for the algorithm was provided by ten Caat et al. [4] and comprises the following steps.

1. A coherence value is assigned to each vertex by calculating the average coherence to all its Voronoi neighbors. All vertices showing local maxima are defined as markers and build the initial functional units.
2. A list containing all edges (i.e., connections) between markers and their Voronoi neighbors is created. The edge with the greatest coherence is the next candidate to be assigned to a marker's unit.
3. After assigning a vertex to a unit, all edges from this vertex to unassigned vertices exceeding the predefined coherence threshold are added to the list.
4. The procedure is repeated until the list is empty.

Finally, coherence between the detected units is calculated as the sum of coherence values between all pairs of electrodes connecting two units divided by the maximal number of connections between the two units. Between-unit coherence indicates the level of connectivity between two units and is considered significant if it exceeds the predefined coherence threshold. This threshold was empirically determined by iteratively applying the clustering algorithm for all thresholds between 0 and 1 with a step of 0.1. For threshold values below 0.3 or above 0.6, the clustering yielded no meaningful results (i.e., only one or no clusters were detected; Supplementary Figure 1). For values between 0.3 and 0.6, we calculated the average number of detected units (i.e., 6; Supplementary Figure S1) and selected the threshold providing this number of units as the predefined threshold for analyses (i.e., 0.5).

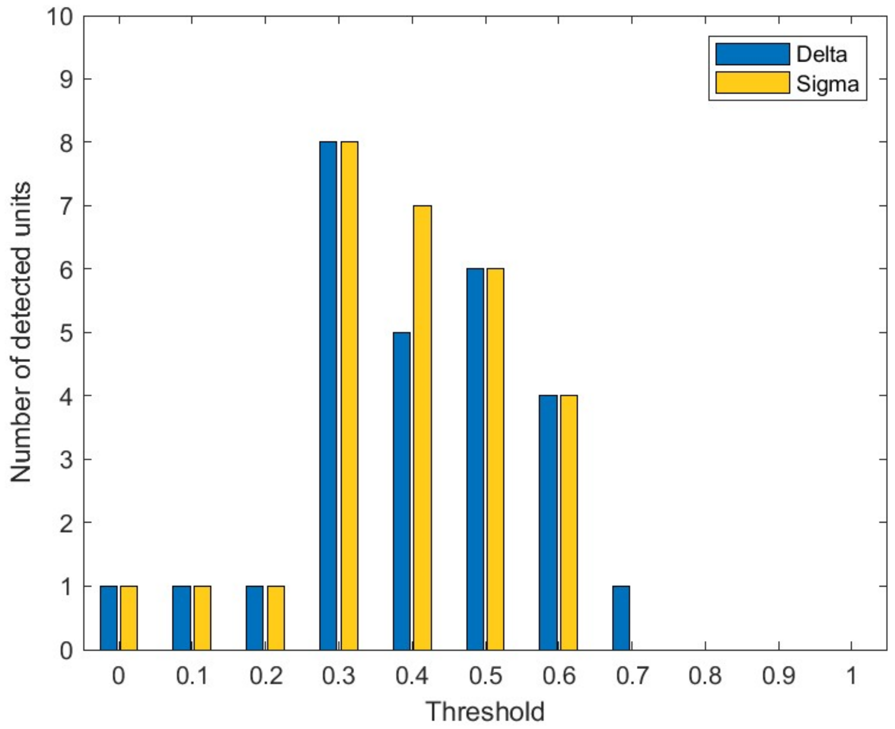

Supplementary Figure S1: The number of functional units detected by the clustering algorithm for different coherence thresholds in the delta and sigma band.

References

1. ten Caat, M. FuMapLab: multichannel EEG Matlab toolbox. (2008).
2. Beucher, S. & Meyer, F. The Morphological Approach to Segmentation: The Watershed Transformation. in *Mathematical Morphology in Image Processing* (ed. Dougherty, E. R.) 433–481 (CRC Press, 2018). doi:10.1201/9781482277234-12.
3. Aurenhammer, F., Klein, R. & Lee, D.-T. *Voronoi diagrams and Delaunay triangulations*. (World Scientific, 2013).
4. ten Caat, M., Maurits, N. M. & Roerdink, J. B. T. M. Data-driven visualization and group analysis of multichannel EEG coherence with functional units. *IEEE Trans. Vis. Comput. Graph.* **14**, 756–771 (2008).
